# Community-based interventions for management of antimicrobial resistance in Europe: a systematic review

**DOI:** 10.1093/eurpub/ckaf257

**Published:** 2026-01-09

**Authors:** Winifred Ekezie, Mayuri Gogoi, Nataly Papadopoulou, Farah Badakshi, Karen J Bowman, Beauty Igein, Manish Pareek

**Affiliations:** Centre for Health and Society, Aston University, Birmingham, United Kingdom; Diabetes Research Centre, University of Leicester, Leicester, United Kingdom; Department of Respiratory Sciences, University of Leicester, Leicester, United Kingdom; Development Centre for Population Health, University of Leicester, Leicester, United Kingdom; Leicester Law School, University of Leicester, Leicester, United Kingdom; Department of Biology, University of Leicester, Leicester, United Kingdom; Health Science Division, Higher Colleges of Technology, Abu Dhabi, UAE; Leicester Drug Discovery and Diagnostics, Institute for Precision Health, University of Leicester, Leicester, United Kingdom; Diabetes Research Centre, University of Leicester, Leicester, United Kingdom; School of Medicine and Population Health, University of Sheffield, Sheffield, United Kingdom; Department of Respiratory Sciences, University of Leicester, Leicester, United Kingdom; Development Centre for Population Health, University of Leicester, Leicester, United Kingdom; Department of Infection and HIV Medicine, University Hospitals of Leicester, Leicester, United Kingdom; NIHR Leicester Biomedical Research Centre (BRC), University of Leicester, Leicester, United Kingdom

## Abstract

Antimicrobial resistance (AMR) is a growing global health problem. Several public interventions have been designed to increase AMR knowledge and awareness. This review assesses the availability and effectiveness of community-based AMR interventions in Europe. Four databases—Medline (OVID), Pubmed, Scopus, Web of Science- and grey literature were searched for AMR interventions in community settings in Europe between 2000 and 2024. Studies reporting empirical findings in English were considered. A narrative synthesis was performed, and findings were presented in text and tables. Forty-nine studies were eligible for inclusion from 14 European countries. Interventions were primarily educational to raise awareness, targeting individuals, small groups, or the general public through mass campaigns, school-based programmes, online games, and pledges. Some interventions also monitored adherence, consumption, and doctor consultation. The majority of interventions reported increased knowledge and awareness of antibiotics and AMR; reduced antibiotic prescription, purchase, use, and non-compliance; reduced respiratory incidence and doctor consultations, and increased overall adherence. Fluctuations in knowledge over time were observed, but evidence was insufficient to analyse the long-term sustainability of outcomes of the interventions. Our findings show that community-based interventions can enhance knowledge and awareness of appropriate antibiotic use and AMR risks among different population groups. These can also positively improve adherence, expectation, and prescribing. However, long-term engagement and interventions are needed to attain sustainability and bring behavioural changes.

## Introduction

Antimicrobial resistance (AMR) is a pressing global problem with several antimicrobials becoming ineffective to treat infections [1]. In 2019, bacterial-resistant infections were directly attributable to about 1.27 million deaths and associated with another 4.95 million deaths globally [2]. In Europe, these figures are 133 000 (directly attributable deaths) and 541 000 (associated with bacterial AMR), respectively [3]. The misuse and overuse of antimicrobials (e.g. non-prescription use, poor adherence, over-prescription) in the community is identified as contributing to AMR [4, 5]. There is, however, variability among European countries in terms of antibiotic consumption within the community. In 2022, Greece reported a mean consumption of nearly 30 defined daily doses (DDDs) per 1000 inhabitants per day in the community, while countries like the Netherlands reported only about 9 DDDs per 1000 inhabitants [6]. Concerningly, compared to 2019, some countries have seen a considerable increase in these consumption rates, most notable being Bulgaria (24.1% increase), Malta (15.7% increase), and Lithuania (13.5% increase) [6]. To tackle AMR, countries in Europe have developed or are developing national action plans and surveillance systems, stewardship campaigns and One Health partnerships. Interventions targeting both the public and healthcare workers have been initiated in several countries [7, 8], and among the general public, educational and behaviour change interventions are the most common [7, 9].

**Figure 1.**
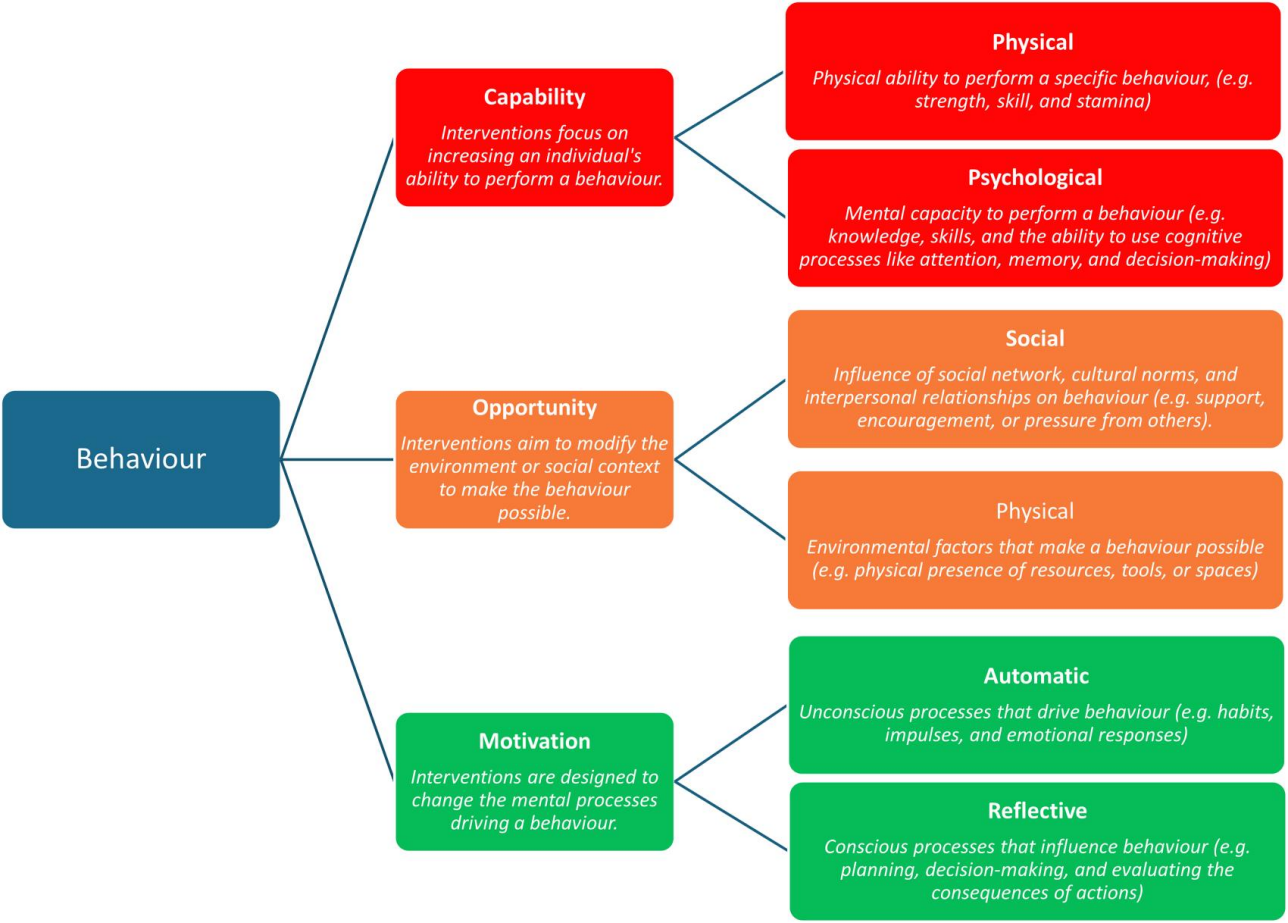
COM-B model.

Previous reviews of AMR public interventions found that interventions targeted at children and parents, and using interactive media, increase knowledge and engagement with AMR stewardship [7, 8]. While these reviews have presented a global picture, focus on community-based interventions (interventions based outside of hospitals/healthcare settings) in Europe is essential due to growing AMR rates in the region [10]. The 2022 Eurobarometer survey, conducted in 27 European Union (EU) member states, found that although antibiotic use had decreased, the majority of respondents (72%) still lacked some essential knowledge about antibiotics, and only half (50%) of respondents knew that antibiotics are ineffective against viruses [11]. Additionally, data also show that third-generation cephalosporin-resistant *Escherichia coli* infections account for the highest burden, and nearly half of these infections occur in the community [1]. This indicates a need for initiating effective community-based interventions, starting with a systematic mapping of evidence on what interventions have been implemented, for whom (which groups), and the outcome of these. We, therefore, conducted this systematic review to assess the availability of community-based AMR interventions in Europe, their reach and effectiveness. Considering that interventions are best supported by evidence-based principles, we have also categorized the interventions according to the COM-B model, a framework for understanding the interaction between Capability (C), Opportunity (O), and Motivation (M), and behaviour (B) (Fig. 1) [12].

## Methods

The review protocol was registered on PROSPERO (CRD42023404108) and followed the Preferred Reporting Items for Systematic Reviews and Meta-Analyses (PRISMA) guidelines [13]. A comprehensive search of peer-reviewed literature was conducted in consultation with a medical librarian.

### Eligibility criteria

Pre-defined eligibility criteria for inclusion included studies reporting (i) primary findings related to AMR community-based interventions (these are interventions based outside of hospital or healthcare settings and aimed primarily at members of the public), (ii) Europe, and (iii) English language from 2000 to date. All study designs were included if they met the inclusion criteria. Systematic reviews, studies only in hospital settings, and studies published before 2000 were excluded.

### Search strategy

We searched MEDLINE (via Ovid), PubMed, Scopus, Web of Science, and the first ten pages of Google Scholar from 2000 to October 2024. Search terms were based on a combination of keywords for three key concepts: ‘Antimicrobial drug resistance’, ‘Community health services and intervention’, and ‘Europe’ (Supplementary Table S1). Reference lists of eligible studies were searched to identify additional studies.

### Study selection

One author (WE) performed the search, and references were uploaded to Covidence review manager. Duplicates were removed, and titles and abstracts of the remaining studies were manually screened by two authors (WE, MG, NP, FB, KJB, BI) independently; discrepancies were resolved through discussions between two authors (WE and MG). Full-texts were screened by two reviewers independently.

### Study appraisal

Quality assessment was conducted by two reviewers (MG and BI) using the Joanna Briggs Institute (JBI) checklist for study designs [14]. Studies were graded as low (>50%), medium (50–70%), or high (<70%) based on how many of the assessment requirements were met.

### Data extraction

Two reviewers independently extracted the relevant data using a structured data extraction tool. Variables extracted included author(s), year, country, study population, design, setting, type of intervention, and reported outcomes. Measures of effect extracted were proportions and confidence intervals, average mean, standard deviations, prevalence ratios, and other applicable measures reported in the included studies. The main themes of the qualitative studies were also extracted.

### Data synthesis

Findings from the included studies were entered into tables and descriptively synthesised following the Synthesis Without Meta-analysis (SWiM) guideline [15]. Two reviewers (WE and MG) read the intervention descriptions to identify the corresponding COM-B category (Fig. 1). Primary outcomes were community-based interventions for managing AMR and their outcomes. Secondary outcomes were intervention target populations and their uptake rates. The analysis explored outcome variations and presents only results for quantitative data. However, due to incomplete and inconsistent measures of effects, a meta-analysis was not conducted.

## Results

### Study selection and characteristics

**Figure 2.**
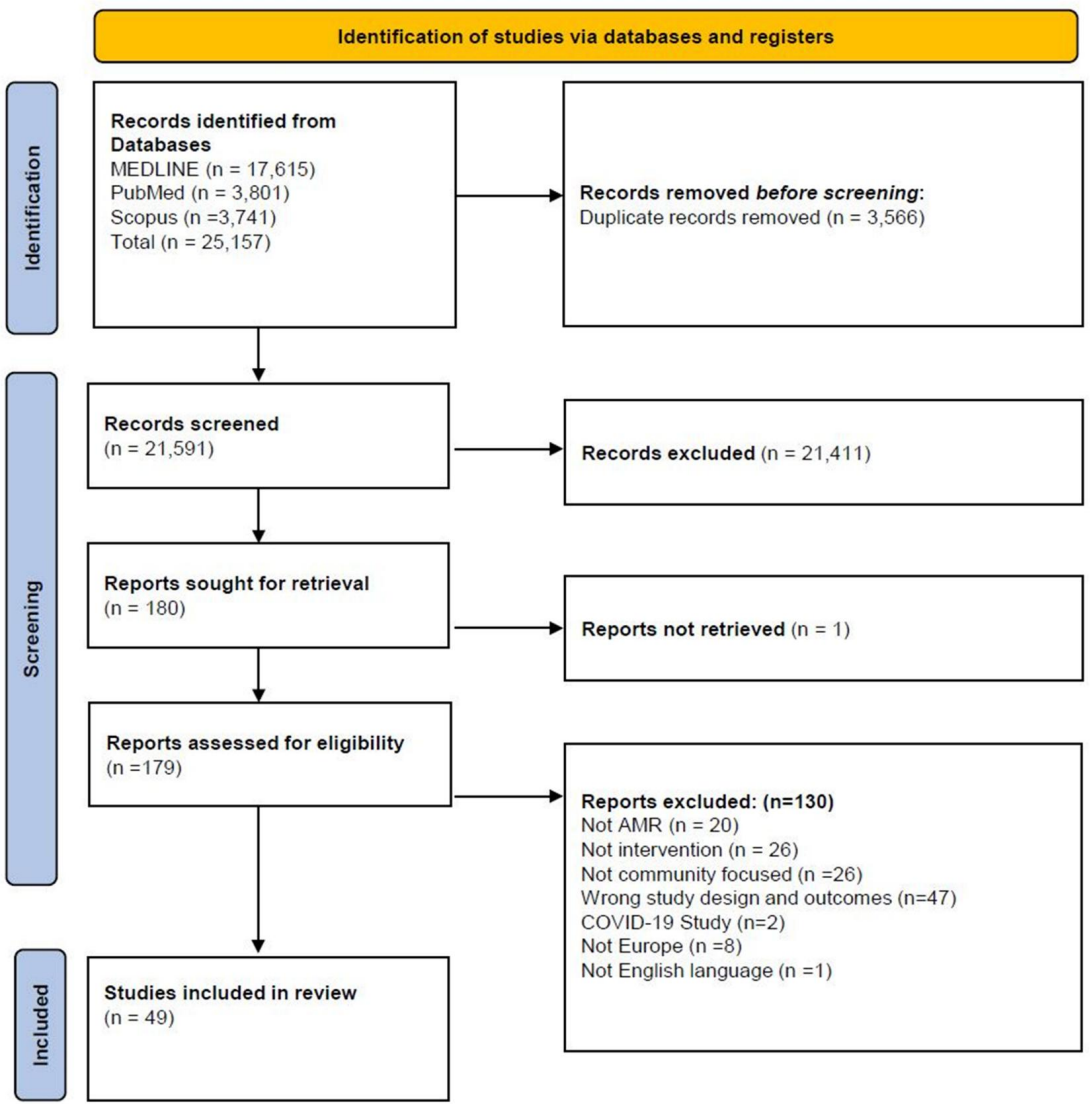
PRISMA flow diagram of study inclusion.

A total of 25 157 search results were identified, 3566 duplicates removed, 21 591 titles and abstracts screened, and 179 full-texts reviewed (Fig. 2). 130 full-text studies excluded had no information on the outcomes or community-related settings, or were conducted during the COVID-19 pandemic, making participation inequitable. Finally, 49 publications were included (Table 1).

**Table 1.** Included study characteristics^a^

Authors	Country	Duration	Design	Sample population	Setting/intervention	Results	Quality
**Ahmed et al. 2020a**	United Kingdom	NR	Quasi-experiment	159 students (15–16 years)	School AMR roadshows (Workshops on handwashing and AMR)	- **Pre-event**: 3 of 11 questions (27%) answered correctly by ≥50% students - **Post-event**: 9 of 11 questions (82%) were answered correctly by ≥50% students; 91% positive increase in knowledge; One question showed a decrease in knowledge - **12 weeks post-event:** 100% positive change	Medium
**Ahmed et al. 2020b**	United Kingdom	2017	Quasi-experiment	Total (*n* = 242) - 137 Think tank museum - 105 Science festival	Science Museum and festival Drama play (e-Bug script)	**Pre- and post-mean self-assessed responses to 8 questions** - Significantly altered scores for Questions 1, 2, 3, 4 and 7 (knowledge questions) (*P* < .05) after presentation in both groups - Significant changes in scores for Questions 5, 6 and 8 (usage questions) only in the Think Tank audiences.	Medium
**Aldeyab et al. 2014 [** 19 **]**	United Kingdom	2006–2010	Quasi experiment	Total (*n* = 2064) - 660 MRSA hospital cases - 1404 MRSA local community cases	Hospital Community Educational (leaflet to high-risk areas)	**Hospital** Revised antibiotic policy intervention not associated with a significant change in MRSA level (*P* = .57) Significant change in trend (*P* = .0057) MRSA incidence rate reduced by 0·00561/100 bed-days per month. **Community** Intervention associated with a significant trend change in MRSA incidence (*P* = .0299) MRSA incidence rate reduced by 0·00004/1000 persons per day. No significant change in MRSA incidence in the community (*P* = .1848)	Low
**Allison et al. 2017**	United Kingdom	2015	Quasi-experiment	NR	University Educational workshops (Antibiotic Guardian pledge)	**Antibiotic Guardians** **Pledges:** 40% pledged, 31% promised to pledge later, 38% did not pledge (many already Guardians) **Knowledge:** Thought antibiotics work against flu (21%), chicken pox (10%), and viruses (17%). - 17% took antibiotics given by a family member, friend or neighbour - 12% thought antibiotic resistance was body adapting to the antibiotic	Low
**Atkins et al. 2020**	United Kingdom	2020	Mixed methods	Total (*n* = 83) - Patients (*n* = 15) - Prescribers (*n* = 22) - Community pharmacy staff (*n* = 8) - Health providers (*n* = 18) - Commissioners (*n* = 18)	Primary and community care Review (of nationally adopted interventions)	***Identified 39 national interventions*** - **Interventions by target group**:- 15 aimed at patients/public (*n* = 15), prescribers (*n* = 22), community pharmacy staff (*n* = 8), providers (*n* = 18), commissioners (*n* = 18) - **Interventions by group coverage:** one target group (*n* = 19), two target groups (*n* = 9), all five target groups (*n* = 3) - 30 BCTs identified across all interventions with the mean number of 4 per intervention - **Patient and prescriber interventions** most commonly ‘information about health consequences’ and ‘instruction on how to perform the behaviour’. - **Pharmacy, provider, and commissioner** most frequently used ‘instruction on how to perform the behaviour’.	Medium
**Azevedo et al. 2013**	Portugal	NR	Quasi-experiment	82 students	School Presentations and discussions	- ***Pre-test:*** School-1 (2.7%), School-2 (0%) correct answers on antibiotic use against bacteria and other organisms - ***Post-test:*** Significant increase in correct answers—School-1 ((*P* = .005) and School-2 (*P* = .001)	Medium
**Azor-Martinez et al. 2018 [** 27 **]**	Spain	2013–2014	RCT	911 children (0–3 years)	Day Care Centres (DCC) Workshop (Hand hygiene)	- ***Antibiotic prescriptions:*** HSG vs CG (IRR: 0.69; 95% CI : 0.57–0.84); SWG vs HSG (IRR: 1.21; 95% CI: 0.1.06–1.39); SWG vs CG (IRR: 0.94; 95% CI: 0.82–1.08) - ***RI episodes and antibiotic prescriptions*** (mean per child per month): HSG vs CG (*P < .05), HSG vs SWG (**P < .05)	High
**Barchitta et al. 2020**	Italy	2018	Cross-sectional	NR	Online internet platforms Interactive website (epidemiological scenario) Social media campaign	- 1159 unique website visitors, 190 pledged campaign champions (conversion rate = 16.4%) - ***Total pledges:*** *n* = 458 [general public (61%), experts (39%)] - Audience aged 18–54 years; 64% female, 36% male - LinkedIn (over 1200 connections), Facebook (675 followers). Instagram (82 followers), Twitter (16 followers) - ***Facebook reach:*** 150 to 12 000	Medium
**Bauraind et al. 2004**	Belgium	2000–2002	Cross-sectional	NR	Mass media campaign	**Antibiotic sales (DDDs)**: - Decrease in 2000–2001 (11.7%), 2001–2002 (9.6%) compared to 1999–2000. - ***Yearly antibiotic sales (DDD)***: 2.9% increase in 1997–1999; 5.3% decrease 2000 - 2002 - Campaign periods associated with overall decrease of 1 354 518 (SD =449 646) in 2000–2001, 1 195 290 (SD, 592072) in 2001–2002	Low
**Bhattacharya et al. 2016 [** 22 **]**	United Kingdom	2014–2015	Cross-sectional (Process evaluation)	NR	Website (Antibiotic Guardian pledge)	**Campaign Reach and Pledges made** - 58 086 website visits by 47 158 unique visitors (81.19%) in 156 different countries - 12 509 became Antimicrobial Guardians (AGs), - At least one pledge in 81 countries (95% in the UK)	Medium
**Bruyndonckx et al. 2021 [** 16 **]**	Belgium	1997–2018	Mixed methods (Survey and Interviews)	NR	National Media campaign	- General practitioners preferred source of improved by 87.5% - Antibiotic use decreased by 12.8% in DID, and 42.8% in PID	Medium
**Cebotarenco and Bush 2008**	Moldova	2003–2004	RCT	3586 students 2156 adults	Schools Peer-led education Parents meetings led by student trainers (STs) Interactive video Booklet Newsletters Poster Poster contest	**Those who had not taken an antibiotic for a cold and/or flu in the previous winter** - ***Students:*** Intervention increased 19.5% to 53.6%; 33.7% relative to the control group - ***Adults:*** Intervention increased 23.0% to 61.1%; 38.0% increase relative to the control group - Males were more likely to say they had not taken an antibiotic for their cold or flu **Those who did not know if they had taken an antibiotic** - ***Student:*** Decreased from 30.0 to 9.2%; 15.1% decrease compared with control group - ***Post-I Students*** more likely not to have taken antibiotics (OR = 3.2 (CI: 2.1–4.9). - ***Post-I Adults*** more likely not to have taken antibiotics (OR = 5.2 (CI: 3.61–7.47)	Low
**Chaintarli et al. 2016 [** 21 **]**	United Kingdom	2016	Cross-sectional (survey)	2478 adults	Online National Campaign (AMR self-reported knowledge and behaviour)	**Change in self-reported behaviour** - Self-reported action in line with their pledge: pre-campaign (30.7%) to post-campaign (63.4%) - Public more likely to act in line with their pledge than professionals (aOR =3.60, CI: 2.88–4.51). - AGs remembered pledge have positive post-campaign behaviour (aOR = 1.96, CI: 1.63–2.36). **Knowledge/awareness of AMR Post-Campaign** - 44.5% acquired more knowledge about AMR - More knowledge if they had not had pre-campaign knowledge (aOR = 4.21, 95% CI: 2.04–8.67) - Public less likely to have acquired more knowledge than healthcare professionals (aOR = 0.79, 95 % CI: 0.66–0.96). - People confused about AMR prior, acquired more knowledge (OR = 3.10, 95% CI: 1.36–7.09) - More had sense of personal responsibility towards tackling AMR (increase from 58.3% to 70.5%)	Medium
**Chan et al. 2021**	United Kingdom	NR	Quasi-experiment	100 respondents	Online/Digital Tailored Logic algorithm (personalised behaviour change messages to address patients’ beliefs)	**Effects on Beliefs (three domains: Necessity, Concerns and Knowledge scores)** Reduction in perceived necessity for antibiotics, increase in concerns, and increase in scores for general perceptions (mean shift in scores: 0.2–0.5 per item) **Scores after the intervention:** - ***Necessity:*** Decreased (67%), Unchanged (22%), Increased (11%). Reduction by 2.29 points (t = 7.254; *P* < .0001) - ***Concerns:*** Increased (53%), Unchanged (40%), Reduced (7%). Mean difference 0.930 (t= −7.214; *P* < .0001); - ***General perceptions:*** Improved (58%), Unchanged (19%) and Declined (23%). Overall perception shift (30%) by 1.08 (t= −4.651; *P* < .0001),	Medium
**Farrell et al. 2011**	United Kingdom	2009	Quasi-experiment	Total (*n* = 1736) - 62 in school students - 1674 online	School Web-based game (Adapted e-Bug pack evaluation test in game show)	Majority did not change knowledge No clear trend in significance of the knowledge change for all five game levels **Questions with most significant knowledge change:** - Question: ‘We use good microbes to make things like bread and yogurt’ (*P* < .001, x2 = 14.46) - Question: ‘If you cannot see a microbe, it is not there’ (*P* = .02, x2 = 5.60) - Question: ‘Soap can be used to wash away bad bugs’ (*P* = .02, x2 = 5.28)	Medium
**Fonseca et al. 2012**	Portugal	2010–2011	Quasi-experiment (pre- & post-test; mixed methods)	Total (*n* = 42)	School (University) Practical (laboratory safety and significance of antibiotic resistance)	**Quantitative: Pre- and Post-test Performance** - Significant differences for every question (*P* < .05). - Improvements in quality of participants’ knowledge **Qualitative: Observation of the Participants** - Misconceptions and difficulties: No relevant difficulties or misconceptions were identified, although several participants admitted that they ‘did not know the human body harbours so many bacteria’ and that ‘antibiotic drugs affect bacteria from the human microbiota’.	Medium
**Formoso et al. 2013**	Italy	2011–2012	Quasi-experiment (non-RCT)	600	Mass campaign (Designed by doctors and pharmacists)	**During the follow-up period:** - ***Average prescribing rates:*** IG (20.0 to 22.7, -11.9% difference), CG (21.0 to 22.7, -7.4% difference) DDD per 1000 inhabitants/day - ***Reduction in antibiotics expenditure compared to previous year***: IG (25.1%), CG (21.8%)	Medium
**Francis et al. 2015**	Italy	NR	Quasi-experiment	NR	Mass media	**Compared with the same period the year before** - ***Antibiotic prescribing reduction:*** Intervention (11.9% reduction - 22.7 to 20 DDD/1000 inhabitants per day) vs control (7.4% reduction; from 22.7 to 21) areas. - ***Prescribing intervention vs control area reduction:*** 4.3% (95% CI: -7.5% to -1.5%) - ***Recall of campaign slogans and graphic:*** Similar in both areas. - Knowledge and attitudes consistent with campaign messages worsened in both areas	Low
**Gilham et al. 2023 [** 29 **]**	United Kingdom	2017–2019	Quasi-experiment (Mixed methods)	For each of 3 waves >1000 public 300 from GPs - pre- campaign questionnaire (public) - post-campaign interview (from GPs)	Mass media Social media	**Knowledge and awareness of AMR** - ***Perceived knowledge AMR improved*** (X2 = 13.952, *P* = .003; X2 = 20.219, *P* = .003; X2 = 20.219, *P* < .001 respectively). - Perceived knowledge of AMR vs antibiotics: Before (23.4% vs. 58%); After (9.2 vs. 8.1%) - Never heard of AMR after campaign (40.5%, 387/956); for antibiotic resistance (7.6%, 74/969) - Parents concern of AMR for children increased by 6.0% (pre-campaign: 53.7%, 495/922); post-campaign (59.7% (751/1258) (Z=−3.616, *P* < .001)] - Parents agreeing to always take doctor’s advice on whether their child needed antibiotics remained stable **Change in reported behaviour** - 5.1% more unlikely to ask for antibiotics after first year of campaign (X2 = 6.067, *P* = .014); After campaign final year returned to pre-2017 measure (pre-2017: 72.6% vs post-2019: 72.0%). - 9.6% increase in parents unlikely to ask for antibiotics for their child after first year (pre-2017: 54.4% (203/373); post-2017: 64.0% (244/381); X2 = 7.645, *P* = .006). After campaign final year reduced to 57.6% (76/132) **General practitioners** - Almost all GPs recognised campaign raise awareness of AMR - Most GPs agreed campaign supported them to say no to patients asking for antibiotics and made patients less likely to ask for antibiotics. [After first year, 2017 = 98.9% (182/184) vs 92.4% (97/105)], X2 = 4.000, *P* = .045); no difference in final year - In 2018, increase in GPs who had seen the campaign stating reasons for antibiotics were inappropriate (90.4% (161/178) vs 75.2% (82/109), X2 = 9.217, *P* = .002); no difference post-2019	Medium
**Hale et al. 2017**	United Kingdom	NR	Quasi-experiment (Mixed methods)	Total (*n* = 153 children) - 153 pupils: pre-and postgame questionnaires. - 135 pupils: qualitative - 48 pupils (focus groups) - 4 pupils in think-aloud sessions.	School (Primary) E-bug games	**Quantitative data** - 2.0% to 13.1% increase in the number of participants answering each question correctly - Significant increase for questions on knowledge about the effectiveness of antibiotics against bacteria and viruses (*P* < .05). - ***Highest knowledge was for the question ‘most coughs and colds get better without antibiotics’***: 68.6% (105/153) correct - ***Lowest knowledge was for the question ‘what antibiotics do’***: 9.2% (14/153) correct. **Qualitative data** Learning experience of doctor was well received. Many of the pupils demonstrated awareness of the topics covered during the gameplay. Generally positive learning experience appeared to accompany Body busters When asked what they had learnt, none of the comments were linked to the learning objectives (‘you must finish the whole course of your antibiotics’, and ‘antibiotics kill bacteria’).	Medium
**Hall et al. 2020 [** 30 **]**	United Kingdom (Scotland)	2009	Mixed methods	182 children (8 - 11-year-olds) 2 primary schools	School (Primary) School musical (developed by composer and an infectious disease doctor)	**Quantitative data** - 180 children completed an online knowledge questionnaire; 6-month follow-up (*n* = 67 children who performed the musical) - ***Covered microbes in school lessons prior to the musical:*** 100% (180/180) - ***Had heard of AMR***: 62% (112/180,). - ***Previous antibiotic use with only reporting:*** Varied previous use; Never received antibiotics (29%, 52/180). - Significant increase in knowledge post-musical in five out of the seven key questions - Two weeks after the musical, 61% (98/161) agreed musical had changed their view of antibiotics; 65% (107/161) enjoyed the experience of performing, 13% (21/161) had not found the musical easy to understand. **Qualitative data** - ***Knowledge of antibiotics and antimicrobial resistance:*** Before the musical, knowledge in some areas was limited. Musical children demonstrated more consistent and improved understanding of antibiotics as well AMR and the factors contributing to this, crucially antibiotic overuse. - ***Behaviour change in relation to infectious diseases and antibiotics:*** Before the musical, some children were able to identify the need to reduce antibiotic use to reduce AMR. Following the musical many children cited motivation to spread awareness of AMR and change behaviour. - ***Engagement of children to spread the message of AMR:*** Before the musical, children reported AMR was not a topic discussed amongst their direct community. After the musical children were able to identify ways, they might be able to involve themselves in this process of engaging with others on a personal and educational level. - ***Unique learning medium:*** Children and teachers felt that the musical was a fun and engaging way to learn about these topics compared to traditional teaching methods. All groups stated there were positive psychosocial benefits to participation such as enjoyment, increased confidence and self-esteem**.**	Medium
**Hedin et al. 2006** [28]	Sweden	Not Reported	Intervention study (Prospective)	Children (*n* = 285) Personnel (*n* = 63) Day Care (*n* = 6)	Day-care centres (DCC) Educational (against infectious diseases)	Intervention: Children (*n* = 140), Personnel (*n* = 31); Control: Children (*n* = 145) Personnel (*n* = 32) - ***Sickness absence days*** [mean (SD)]: IG [10.5 (8.6)], CG [11.2 (7.4)]; RR: 0.95 (95% CI: 0.78 - 1.15) - ***Doctor consultations per child*** [mean (SD)]: IG [0.8 (1.2)], CG [1.1 (1.3); RR : 0.81 (95% CI: 0.63 - 1.04) - ***Antibiotics prescription per child*** [mean (SD)]: IG [0.4 (0.8)], CG [0.7 (1.0)]; RR: 0.70 (95% CI: 0.48 -1.02) - ***Percentage given antibiotics***: IG (38%), CG (42%) No statistically significant effect	Medium
**Hogberg et al. 2004**	Sweden	2001–2002	Mixed methods (survey, laboratory data, cohort)	Children (*n* = 224) Day care (*n* = 25) - Intervention: (14 DCCs, 213 children) - Control: (11 DCCs, no children sample)	Day-care centres (DCCs) Surveillance and Lab Testing (reduce rate of AMR carriage)	Intervention—[Area A (11 DCC, 165 children), Area B (3 DCC, 48 children)] - ***Before intervention***: Area A (11 index cases, 26 children) Area B (3 index cases, 10 children) - ***Follow-up period***: Area A (4 children), Area B (7 cases) - ***Incidence of PNSP after intervention period***: Area A [2.9% (4/139)], Area B [18.4% (7/38)]; RR: 6.4; 95% CI: 2.0 - 20.7). - ***Attributable fraction (proportion new carriers estimated to be attributed to the lack of intervention) during the follow-up period in study area B***: 84% (95% confidence interval: 49/95).	Medium
**Kesten et al. 2017**	United Kingdom	2015–2016	Qualitative	48 respondents	Online (Antibiotic Guardian (AG) Website)	- ***Campaign awareness:*** Most AGs struggled to remember where they had heard about the campaign - ***Campaign decision making***: Majority of AGs were motivated to join the campaign by their pre-existing interest and concern about AMR related issues. - ***Pledge group and pledge choices:*** Several AGs were eligible for more than one pledge group but the website asked them to select one. - ***Pledge recall:*** Despite an automated email and certificate being sent to all AGs thanking them for participating in the campaign and listing their pledge, the majority could not recall which pledge. - ***Fulfilling the pledge***: The majority of AGs felt they had fulfilled their pledge. Extent of fulfilment attributed to factors such as: pledges matching pre-existing behaviour; not having an opportunity to fulfil the pledge and; consciously acting in line with pledges (e.g. promoting the campaign to others). - ***Raising and reinforcing awareness:*** Mixed reports of the campaign informing AGs about AMR. Some AGs described learning new information including the scale of the AMR issue while others had learnt little and felt the campaign was a continuation of their current thinking.	High
**Lecky et al. 2010**	Czech Republic England France	NR	Quasi-experiment	2847 students Junior students (JS) - Czech Republic (IG: 191, CG: 223) - England (IG: 357, CG: 57) - France: (G: 233, CG: 136) Senior students (SS) - Czech Republic (IG: 263, CG: 250) - England (IG: 583, CG: 110) - France (IG: 250, CG: 194)	Schools E-Bug teaching pack (taught in specific sections of country’s National Curriculum)	Post intervention knowledge change (95% CI) IG vs CG (Note: London has JS and SS control group) **Introduction to Microbes** - ***Czech Republic***: Prague: JS (IG: 26.4, CG: 14.1), SS (IG: 13.8, CG: 14.8); Ostrava: JS (IG: 30.8, CG: 2.1), SS (IG: 24.0, CG: 0.8) - ***England:*** Gloucestershire: JS (IG: 32.5, CG: 33.5), SS (IG: 18.9, CG: 12.3), London: JS (IG: NA, CG: NA), SS (IG: 14.9, CG: NA) - ***France:*** Nice: JS (IG: 36.4, CG: 29.1), SS (IG: 20.3, CG: 19.1); Bordeaux: JS (IG: 36.4, CG: 29.1), SS (IG : 22.2, CG: 20.3) **Spread of Infection** - ***Czech Republic:*** Prague: JS (IG: 9.4, CG: 3.4), SS (IG: 14.2, CG: 7.0); Ostrava: JS (IG: 8.6, CG: -5.3), SS (IG: 16.5, CG: 2.2) - ***England:*** Gloucestershire: JS (IG: 10.5, CG: 10.6), SS (IG: 15.0, CG: 7.0); London: JS (IG: NA, CG: NA), SS (IG: 13.2, CG: NA) - ***France:*** Nice: JS (IG: 6.2, CG: -7.7), SS (IG: 14.0, CG: 10.8); Bordeaux: JS (IG: 15.5, CG: 11.5), SS (IG: 14.9, CG: 12.2) **Treatment and Prevention of Infection** - ***Czech Republic:*** Prague: JS (IG: 29.8, CG: 19.8), SS (IG: 33.2, CG: 30.5); Ostrava: JS (IG: 28.3, CG: 10.1), SS (IG: 43.4, CG: 14.1) - ***England:*** Gloucestershire: JS (IG: 15.4, CG: 16.8), SS (IG: 24.4, CG: 14.3); London: JS (IG: NA, CG: NA), SS (IG: NA, CG: 16.2) - ***France:*** Nice: JS (IG: 22.5, CG: 24.1), SS (IG: 33.1, CG: 28.1); Bordeaux: JS (IG: 30.8, CG: 26.9), SS (IG: 26.1, CG: 34.1)	High
**Lecky et al. 2017**	United Kingdom	2012	Quasi-experiment	NR	Hospital Animations and Poster campaign	**Intention to change treatment behaviour** (Next time they have a flu, cough, cold or sore throat) - 59.8% less likely to see a GP (*P*= .001). - 63% less likely to ask GP for antibiotics (*P*< .001) - 54.6% more likely to drink plenty of fluids (*P*= .01) - 65.9% more likely to rest (*P*< .001) - 59.8% more likely to take paracetamol (*P*< .001) - 13.0% less likely to take antibiotics without the recommendation of a doctor or nurse (*P* = .3) - ***Carers views:*** Only 29.5% with young children would less likely to ask for antibiotics the next time their child (<5 years) had a flu, cough, cold or sore throat (*P* = .01) and 61.2% would be neither more or less likely	Medium
**Madle et al. 2004**	United Kingdom	2003	Quasi-experiment (pre- and post-intervention)	177 museum visitors	Science Museum AMR information on website	**Changes in knowledge about antibiotics and AOM** (acute otitis media) - ***Response to ‘people cannot become resistant to antibiotics’***: 46% to 10%; 36% change (*P* = .001, χ2 = 60.357, 95% CI: 27.47–44.53) - ***Response to ‘antibiotics do not cure most sore throats’:*** 57% to 75%; 18% change (*P* = .001, χ2 = 19.22, 95%CI 8.62–27.38). **Changes in attitudes to prescribing antibiotic use in AOM** - ***Believed ‘Doctors should usually prescribe antibiotics for a child with AOM’***: 51% to 33%; Mean: 3.33 to 2.84, –0.49 change (*P* < .001, 95%CI: –0.72 to –0.26) - **Agreement with ‘I would expect an antibiotic for me/my child if I/they had AOM**’: 59% to 30%; Mean: 3.44 to 2.88, –0.56 change (*P* = .001, 95%CI: –0.78 to –0.33)	Medium
**Mazińska et al. 2017** [24]	Poland	2009–2011	Cross-sectional	Adults ≥ 18 years (*n* = 5004)	Telephone based National campaigns (raise awareness on use of antibiotics)	**Response to EAAD campaign in 2009 and 2011** (% Difference, *P*-value) **Use of antibiotics (previous antibiotic exposure)** - ***Limited the use of antibiotics:*** 27% to 43% (16%, *P* = .009) - ***More cautious use of antibiotics:*** 4% to 16% (+12% *P* = .001). - ***Used antibiotics to treat:*** Cold [30% to 21% (-9%, *P* = .008)], sore throats [24% to 12% (-12%, *P* < .001)], flu [16% to 13% (-9%, *P* = .124)] - ***Taken the full dose of antibiotic prescribed:*** 75% to 81% (+6%, *P* = .063) - ***Expected a prescription for antibiotics for:*** colds [19% to 15%, (-4%, *P* = .019)], flu [43% to 32%, (-11%, *P* < .001) - ***Behaviour after receiving EAAD campaign information:*** 38% to 48%, (10% *P* = .012) - ***Knew that imprudent use of antibiotics leads to antimicrobial resistance:*** 86% - ***Believed that antibiotics are effective against:*** flu (49%), colds (36%) - ***By gender:*** More males than females believed effectiveness against cold (OR = 2.16, 95% CI = 1.88–2.48) and flu (OR = 2.2, 95% CI = 1.91–2.54). - ***By age:*** More by younger respondents between the age of 18–24 than older ones (e.g. flu respondents ≥ 60 OR = 0.19, 95% CI = 0.13–0.28) - ***By education:*** Lower education compared to those with higher level (e.g. flu OR = 3.53, 95% CI = 2.62–4.76).	Medium
**McNulty et al. 2001**	United Kingdom	NR	Quasi-experiment before and after	48 children (9 to 11 years old)	School Workshops Site visit to Severn Trent waterworks	**Change in knowledge** - **Knowledge of antibiotics**: 45% to 73% (*P* < .0001). - ***Responds to ‘Where are bugs found?’:*** 80.5% to 93.2% (*P* = .0002). - ***Responds to antibiotic resistance***: 19% to 27% (*P* = .7) - ***Responds to ‘How do bacteria spread?’***: 11% to 29% (*P* < .0001) - ***Responds to ‘When do you need to wash your hands?’:*** 13% to 21% (*P* = .54)	High
**McNulty et al. 2010 [** 31]	United Kingdom	2008 - 2009	Before and after survey (using controls)	Before campaign (2008) *n* = 1888 adults England = 1706 Scotland = 182 After campaign (2009) *n* = 1830 adults England= 1707 Scotland= 123	Community Campaign Posters in magazines, newspapers, patient advice leaflet	**Change in Knowledge and attitudes** - ***Incorrect response to ‘antibiotics work on most coughs and colds’***: England (40% to 37%, *P* = .3), Scotland (40% to 44%, *P* = .6); *P* = .3 - ***Agree ‘If taken too often antibiotics are less likely to work in the future’:*** England (15% to 16%, *P* = .8), Scotland (10% to 10%, *P* = 1.0); *P* = .1 - ***Agree with the statement ‘Resistance to antibiotics is a problem in British hospitals’:*** England (30% to 37%, *P* = .6), Scotland (27% to 29%, *P* = .8); *P* = .5 **Change in Antibiotic use** - ***Reported antibiotic prescription:*** England (34% to 35%, *P* = .7), Scotland (29% to 35%, *P* = .4); *P* = 1.0 - ***Taken antibiotics without being told to do so:*** England (8.3% to 7.8%, *P* = .8), Scotland (3% to 3%, *P* = .8); *P* = .04 - ***Asked GP or nurse for antibiotics in the past year:*** England (28% to 29%, *P* = .7), Scotland (26% to 34%, *P* = .2); *P* = .3 **Change in Recollection of information** - ***Recall of any of the three campaign posters:*** 19.2% to 23.7% (*P* = .03) - ***Recall of the ‘hand’ poster*** (featuring a handful of antibiotic capsules with the slogan ‘Unfortunately, no amount of antibiotics will get rid of your cold’): England (11.6% to 29%, *P* = .01), Scotland (8% to 10%, 0.72); *P* = .19 - ***Recall of general practice patient leaflet*** (in 2019): 5.2% England, 6% Scotland (*P* = .84)	Medium
**McNulty et al. 2020**	United Kingdom	NR	RCT	232 students 5 schools Cardiff Intervention (*n* = 136), Control (*n* = 50) Manchester Intervention (*n* = 21) Control (*n* = 25)	Schools AMR Teaching sessions (e-Bug antibiotics) PE lesson plan	5 schools (3 intervention, 2 control). - ***Volunteer peer-educators:*** 59% (10/17) university students, 28% (15/54) biology students - ***Completed all questionnaires:*** 30% (38/127) intervention, 55% (66/120) control - ***Antibiotic use from GP medical records:*** (54/136, 40% of students’ data available), student SMS (69/136, 51% replied) and questionnaire, (109/136, 80% completed); good agreement between GP and SMS (kappa = 0.72), poor agreement between GP and questionnaires (kappa = 0.06) - Median knowledge scores higher post-intervention, greatest for non-biology students	Low
**Munoz et al. 2014 [** 26 **]**	Spain	2010	Quasi-experiment	126 patients - Intervention (*n* = 64) - Control (*n* = 62)	Community pharmacy Educational (patient antibiotic adherence)	- ***Antibiotic adherence:*** IG (67.2%), CG (48.4%); CI =15.8–34.6, *P* = .033 - ***Non-compliance*** (missing >1 dose): IG (38.1%), CG (81.2%); CI = 16.4—63.1%, *P* = .001] - ***Level of knowledge of antibiotic treatment:*** No knowledge (IG (51.6%), CG: 41.9%), Insufficient (IG: 4.7%, CG: 8.1%), Sufficient (IG 40.6%, CG: 43.5%), Optimum (IG 3.1%, CG: 6.5%,); *P* = .58 - No significant differences in patient-perceived health. - ***Predictor of adherence:*** medication knowledge, coincidence between duration of treatment indicated by physician, duration of treatment written in the prescription.	High
**Newitt et al. 2018 [** 23 **]**	United Kingdom	2016	Cross-sectional	179 239 unique visitors	Website Mass media	- ***AG website visits***: 221 226 times by 81% unique visitors (179 239) - ***Coverage***: Pledges were received from 129 different countries - 492 unique visitors to the Russian webpage, 1124 to the Dutch webpage and 152 to the French page, equating to, adjusted conversion rates of 10.2, 27.3 and 6.7%, respectively. - ***Pledges:*** 42 457 English pledges, 367 non-English pledges (50 Russian, 307 Dutch, 10 French pledges) - ***Overall adjusted conversion rate:*** 23.9%	Medium
**Newitt et al. 2019**	United Kingdom	2014–2017	Cohort (Retrospective)	NR	Website campaign	- **Coverage:** 231 460 unique website visitors from 202 countries - **Visitors:** UK (80.9%), outside of the UK (19.1%) - Top 5 countries visiting the website outside of the UK: United States (*n* = 7391), Russia (*n* = 4675), Belgium (*n* = 3071), India (*n* = 1496), and Australia (*n* = 1420). - **Pledges:** 57 627 English, 652 foreign language pledges. - 627 foreign language pledges and received the following conversion rates; Russian webpage 8.5%, Dutch webpage 19.3% and French webpage 8%. - **Access route*:*** Self-direction route (55%) with pledges more likely than social media (OR 2.6, 95% CI 2.5–2.6)	Low
**Parsons et al. 2004**	United Kingdom	1999–2000	RCT	1000	Hospital Campaign (Mass media and Health Visitors)	CATNAP (Campaign on Antibiotic Treatment and the National Advice to the Public) - ***Response rates in questionnaire study:*** 45 % (442/982) initially, 42% (8191941) at follow-up - ***Believe children should be prescribed antibiotics for fever*** decreased from 56% to 49% - ***Rate of change in prescriptions*** dispensed between 1998/9 and 1999/2000 was not significantly **Change in proportion agreeing that** (initial vs follow-up; difference (95% CI): - ***Antibiotics will help a cough to get better more quickly:*** 42% to 38%; -4% (-9.1%) - ***Antibiotics will help tonsillitis to get better more quickly:*** 77% to 76%; -1% (-5.5%) - ***Antibiotics will help other sorts of sore throats to get better more quickly***: 49 to 49%; -1% (-5.8%) - ***Antibiotics will help a cold to get better more quickly:*** 26% to 23%; -4% (-8.1%) - ***Antibiotics help fight all infections including viruses:*** 51% to 49%; -2% (-6.7%) - ***They should be able to ask their GP for antibiotics by phone***: 27% to 27%; -1% (-5.0%) - ***I should be able to question my GP if they prescribe antibiotics and I do not think they are needed:*** 94% to 90%; -4% (-6.3%)	Medium
**Plachouras et al. 2014**	Greece	2009–2010	Quasi-experiment	293 parents	School Primary healthcare centres Public campaign (press-conference)	772 parents participated; 293 completed questionnaires - ***Antibiotic consumption*** unchanged at January-February (26 DID) increase to March (32 DID) - ***Utilization of Amoxycillin and Penicillin*** increased by 34.3% - ***Use of other antimicrobial classes*** including macrolides, cephalosporins and fluoroquinolones decreased by 6.4%–21.9%.	Medium
**Pos-Doering et al. 2020**	Germany	2018–2019	RCT (Mixed methods)	114 participants Baden Wuerttemberg (BW) region *n* = 60, Mecklenburg Western Pomerania region (MV) *n* = 54	Hospital Multi-media awareness campaign (Educational quality improvement program)	Survey waves—April to July (T1), and March to April 2020 (T2) General Practitioners (GPs), Medical Assistants (MAs) and Paper-based (PBs) components T1 (185 participants: intervention = 41 GPs, 50 MAs) T2 (127 participants: intervention = 32 GPs and 32 Mas, control = 31 GPs and 32 MAs) **Uptake changes reported by:** - GPs: 20 to 88% in T1; 31 to 63% in T2 - MAs: 22 to 70% at T1 and 6 to 69% at T2 - PBs: 64 to 90% in T1; 41 to 93% in T2	Low
**Rawson et al. 2018**	United Kingdom	2015–2016	Qualitative (Focus Groups)	30 past antibiotics patients	Outpatient workshops (AMR knowledge and understanding)	15/18 (83%) participants ***Pre-intervention:*** patients reported desiring further information regarding their infections and antibiotic therapy, including side effects of treatment. ***Post-intervention***: - Improved short term knowledge and understanding of individuals infections and antibiotic management [Median (IQR) scores from 3(2–5)/13 to 10(6–11)/13] - Reported usefulness of intervention (Median (range): - Would participants use the intervention again (*n*, %): 13/15(87%)	High
**Rönnerstrand et al. 2015** [25]	Sweden	2013	Experimental	981 adults ≥ 18 years	Web-based Hypothetical scenarios query (decision mailing process)	- ***Willingness to postpone antibiotic treatment*** [group days (%, mean days (95%CI)]: 1 day [67%; 3.31 (95% CI: 3.05–3.57)], 3 days [67%, 3.74 (95% CI: 3.49–4.00)], 5 days [67%, 4.32 (95% CI: 4.05–4.58)], *P* < .001 (higher in the group in good health, as compared to the group in poor health) - ***Difference in mean willingness:*** (F (2733) 14.575, *P* < .001, h2 = 0.038).	Medium
**Roope et al. 2020**	United Kingdom	2016 - 2017	Experimental (survey)	4000 participants	Online/Digital intervention Three different messages about antibiotics and AMR.	Version 1 (Fear message only, *n* = 1000), Version 2 (Mild fear plus empowerment, *n* = 1500), Version 3 (Strong fear plus empowerment, *n* = 1500) - ***‘Less/much less likely’ to visit a doctor:*** ‘fear-only’ (29.2%), ‘mild-fear-plus-empowerment’ (45.1%), ‘strong-fear-plus-empowerment’, 46.1%); (*P* < .0001), - ***‘More/much more likely’ to visit a ‘doctor’:*** ‘fear-only’ (14.1%), ‘mild-fear-plus-empowerment’ (10.3%), ‘strong-fear-plus-empowerment’, 11.3%); (*P* = .01), - ***‘Less/much less likely’ to ask for antibiotics:*** ‘fear-only’ (42.3%), ‘mild-fear-plus-empowerment’ (52.5%), ‘strong-fear-plus-empowerment’, 54.7%); (*P* < .0001) - ***‘More/much more likely’ to ask for antibiotics:*** ‘fear-only’ (10.1%), ‘mild-fear-plus-empowerment’ (8.2%), ‘strong-fear-plus-empowerment’, 7.5%); (*P* = .08)	Medium
**Roque et al. 2016 [** 20 **]**	Portugal	2014	Pragmatic cluster-randomized trial	25 outpatient centres (∼ 309 physicians) 144 community pharmacies (∼312 pharmacists)	Municipal data review Educational (using distribution of posters, flyers and scientific papers)	**- *Change between baseline to post-intervention:*** decrease in total consumption of antibiotics was observed in both groups Reduction by: IG (0.18 PID), CG (0.09 PID) - ***Overall consumption of antibiotics*** (3.71% decrease, 95% CI: 0.00–8.30) - ***Tetracyclines consumption:*** (15.63% decrease, 95% CI: 2.94–27.59) - ***Macrolide consumption:*** (9.37% decrease, 95% CI: 2.21–17.43) - ***Cephalosporins consumption:*** (7.24% decrease, 95% CI : 0.00–15.80). - ***Penicillin, Sulfonamides, trimethoprim, Quinolones:*** no statistically significant changes	High
**Sabuncu et al. 2009 [** 18 **]**	France	2000–2007	Quasi experiment	453 407 458 individual data record from general population - No control group	Nationwide campaign	- ***From 2000 to 2007:*** Antibiotic consumption declined in all regions between 2001–2002 and 2006–2007 - In 2000–2001, 15/22 regions had .70 prescriptions per 100 inhabitants, but none exceeded this level in 2006–2007. - ***Antibiotic prescription*** [per 100 people baseline in 2000–2002; prescription (% change in 2006–2007)]: All [72.4; 56.6 (-21.9)], Penicillin [27.0; 20.2 (-25.3%)], Cephalosporins [16.3; 12.3 (-24.6%)], Macrolides [16.4; 11.5 (-30.1%)], Quinolones [4.2; 4.7 (+12.8%)], Cycline [3.1; 3.0 (-3.7)] - ***Children <6 years*** (per 100): 2001–2002 (193.3) to 2006–2007 (128.7), [Difference: 64.6] - **Children 2–3 years** (per 100): 2001–2002 (∼250) to 2006–2007 (∼150), [Difference: ∼100] - Substantial reduction of antibiotic use was also observed for children 6–15 y old and young adults 26–35 y old.	High
**Scalas et al. 2017**	Italy	2011–2015	Quasi-experiment	956 students (9–11 years)	Schools Workshops (Adapted e-bug resources)	**Proportion of correct answers (Pre- vs Post- activities)** - ***Antibiotics action (effective against bacteria):*** 48 (5.0%) vs 738 (77.2%); *P* < .0001 - ***Antibiotic use (Unnecessary use of antibiotics can increase bacteria resistance)***: 116 (12.1%) vs 704 (73.6%) *P* < .0001	Medium
**van Hecke et al. 2019** [17]	United Kingdom	2016–2017	Qualitative (Semi-structured interviews)	23 care-giver of preschool children (≤ 5 years)	Homes or places of preference Public awareness campaigns	- Theme 1. Parents’ understanding of antibiotic resistance - Theme 2. Perceived consequences of antibiotic resistance - Theme 3. Ways to reduce antibiotic resistance in response to campaign materials - Theme 4. Parents’ reflections on current antibiotic awareness materials - Theme 5. Social responsibility to inform parents about antibiotic resistance	High
**van Rijn et al. 2019**	Netherlands	2016	RCT	2037 participants	Web-based informational video	− ***General awareness of antibiotic resistance*** (*P* = .048): IG (M = 4.10, SD = 0.42), CG (M = 4.05, SD = 0.44), t (1778)=−2.351, *P* = .019	Low
**West and Cordina 2019**	Malta	2016–2017	Quasi-experiment	Patients (*n* = 400) Pharmacies (*n* = 14) - Intervention (*n* = 200) - Control (*n* = 200)	Community pharmacies Education (adherence and counselling)	- ***Non-adherence:*** Intervention group (10%), control (24%) (*P*≤ .0005) - ***Non-adherent patients with >2 tablets/capsules left:*** IG (70.0%, *n* = 14), CG (79.2%, *n* = 38) - ***Percentage cost of wasted antibiotics:*** IG (3.6%, *n* = 22), CG(10.1%, *n* = 20), 2.8-fold more - ***General-Benefit beliefs (mean ± SD):*** IG(14.80 ± 2.09), CG (14.34 ± 2.44); *P* = 0.044 - ***General-Harm beliefs (mean ± SD):*** IG (11.05 ± 2.12), CG (10.74 ± 2.44); *P* = .176 - ***General-Overuse beliefs (mean ± SD):*** IG (11.88 ± 2.69), CG(11.97 ± 2.79); *P* = 0.743 - ***‘General-overuse’ beliefs:*** higher in CG significantly associated with non-adherence (*P* ≤ .0005).	High
**Wilding et al. 2021**	United Kingdom	2019	RCT	Time 1: (*n* = 479) Time 2: (*n* = 417)	Website Animation film	**Knowledge** - Some ‘good’ bacteria are important for health (M, SD): Time 1 [IG (4.75, 0.40), CG (4.48, 0.56)], at 6 weeks [IG (4.65, 0.49)) CG (4.61, 0.47)]; *P* < .001 - Antibiotics kill ‘good’ bacteria (M, SD): Time 1 [IG (4.44, 0.79), CG (3.77, 0.85), at 6 weeks [IG (4.31, 0.78), CG (4.06 (0.76)]; *P* < .001. - Taking antibiotics when not needed can harm health (M, SD): Time 1 [IG (4.56, 0.50), CG (4.41, 0.55), at 6 weeks [IG (4.56, 0.48), CG (4.52, 0.48); **P < .01 **Attitudes/beliefs** - ***It is best to avoid taking antibiotics unless recommended by my doctor/dentist*** (M, SD): Time 1 [IG (4.77, 0.45), CG (4.63, 0.59)], 6 weeks [IG (4.64, 0.61), CG (4.60, 0.65)]; *P* < .01 - ***I should not expect a doctor or dentist to prescribe antibiotics if they feel I do not need them*** (M, SD): Time 1 [IG (4.55, 0.68), CG (4.39, 0.85)], at 6 weeks [IG (4.47, 0.73), CG 4.45 (0.75)]; *P* < .05 **Intentions** - ***I will not ask my doctor or dentist for antibiotics if I could do without*** (M, SD): Time 1 [IG (4.37, 0.84), CG (4.15, 1.00)], at 6 weeks [IG (4.21, 0.89), CG (4.31, 0.76)]; *P* < .05 - ***I plan to avoid treating myself with antibiotics*** (M, SD): Time 1 [IG 4.38, 1.09), CG [IG (4.26, 1.13), at 6 weeks [IG (4.43, 0.97), CG (4.20, 1.23)]; *P* < .05	Low
**Young et al. 2017**	United Kingdom	2013–2014	Quasi-experiment (Mixed methods)	476 students (4 schools)	Schools Teaching/Peer educator led workshop (E-Bug resources)	1576 questionnaires completed (before, after and retention questionnaires) **Students’ pre-knowledge** - 4 of 5 topics had highest pre-knowledge in the inner-city school; lowest in all topics in the rural schools. - Hand hygiene topic pre-knowledge highest amongst both primary and secondary schools; antibiotics knowledge lowest. - Significant improvement in knowledge for each topic was observed in both primary and secondary school students. - Knowledge improvement greatest in the rural schools and lowest in the inner-city school. - Knowledge at 6 weeks post-intervention higher than before the workshop - Significant decline in knowledge in the antibiotic topics in the primary rural schools - In some regions and topics, knowledge in the retention questionnaire actually increased, e.g. the primary town and inner city schools for the Microbe Mania topic. **Peer-educators** - Did not have a higher baseline knowledge compared with non-peer educators - No notable difference in peer/non-peer knowledge change in the after questionnaire. - Higher level of knowledge retention compared with non-peers for some topics - This is particularly evident in the antibiotics topic, where peer-educators’ knowledge actually **Qualitative data** - ***Five main themes from teacher interviews:*** reasons for running the intervention; planning and organization of the intervention; future of the intervention; student knowledge gain; and student behaviour change. - ***Four themes from the student focus groups:*** expectations prior to the intervention; knowledge and behaviour change; teaching others; and planning and running the event.	High
**Young et al. 2019**	United Kingdom	2016	Quasi-experiment: Mixed methods	235 students (13–16 years) 5 teachers	Schools Educational lessons and Debate (e-Bug Antibiotic Resistance debate lesson)	- ***Antibiotics can cure the common cold:*** Before [80.0%; OR(CI): 1.0), After [96.2%, OR(CI): 7.34 (3.13–17.2)]; *P* < .001 - ***Antibiotics do not harm ‘good’ bacteria:*** Before [64.3%; OR(CI): 1.0), After [87.2%, OR(CI):6.90 (3.44–13.8)]; *P* < .001 - Only certain type of bacteria can become resistant: Before [48.1%; OR(CI): 1.0), After [80.4%, OR(CI):16.2 (6.57–40.0)]; *P* < .001 - ***Antibiotics can be used as painkillers:*** Before [51.1%; OR(CI): 1.0), After [80.4%, OR(CI):5.90 (3.35–10.4)]; *P* < .001 - ***The more we use antibiotics, the more antibiotic resistant microbes appear:*** Before [87.2%; OR(CI): 1.0), After [93.2%, OR(CI):2.40 (1.15–5.03)]; *P* = .02 - ***Microbes can only be resistant to one type of antibiotics:*** Before [72.3%; OR(CI): 1.0), After [85.5%, OR(CI): 3.23 (1.77–5.90)]; *P* < .001	High

a Reference numbers presented for only studies cited within the main paper. Full list of included study references is shown in Supplementary Table S2. AMR, antimicrobial resistance; CG, control group; IG, intervention group; CI, confidence interval; DDD, defined daily doses; OR, odd ratio; PID, packages per 1000 inhabitants per day.

### Characteristics of included studies

The included studies represented 14 European countries, mostly from the United Kingdom (UK) (*n* = 27 studies) (Table 1). Interventions were conducted between 2002 (France) and 2020 (UK), spanning several months to up to 21 years [16]. Studies represented 453 630 783 participants (range: 23–453 407 458) [17, 18]. The study locations included homes, community centres or community pharmacies, daycare centres, schools and universities, online (web-based) platforms, hospital outpatient services, and the use of national public records. The specific study target populations included the general public, students, parents, daycare centre staff, and healthcare providers (including physicians, pharmacists, prescribers, and commissioners). Disaggregated sociodemographic data for those reached by the intervention, such as age, gender, ethnicity/race, education, family size, or social class, were not reported in most studies.

Various intervention study designs and combinations were used, primarily quantitative studies, including cross-sectional (survey and large data reviews) and experimental (using randomization). In addition, there were 10 mixed-methods and three qualitative studies. Overall study quality was medium (11 low-quality, 27 medium-quality, and 11 high-quality). Differences in study quality were mainly related to outcomes measurement validity and reliability. Considering the significant heterogeneity across the studies, an in-depth statistical analysis of the findings was not feasible.

### Types of AMR community interventions

The most common element of behaviour change addressed in the majority of interventions was psychological capability (*n* = 48 studies) (Table 2). Interventions were primarily educational, but approaches used differed by population scale, i.e. specific individuals, small groups, or the general public (Table 2). Having physical opportunities to practice skills, such as practical workshops, was also common (*n* = 15 studies). Test of physical capabilities and automatic motivation from unconscious processes were the least approaches (*n* = 7 and *n* = 8 studies, respectively).

**Table 2.** Matrix of interventions COM-B^a^

Component	Capability		Opportunity		Motivation	
Model of behaviour	Physical	Psychological	Physical	Social	Reflective	Automatic
Ahmed et al. 2020a	✓	✓	✓			
Ahmed et al. 2020b		✓				
Aldeyab et al. 2014 [19]	✓	✓	✓	✓		
Allison et al. 2017		✓	✓	✓	✓	✓
Atkins et al. 2020		✓				
Azevedo et al. 2013		✓			✓	
Azor-Martinez et al. 2018 **[**27**]**	✓	✓	✓			
Barchitta et al. 2020		✓		✓		
Bauraind et al. 2004		✓				
Bhattacharya et al. 2016 **[**22**]**		✓	✓	✓	✓	✓
Bruyndonckx et al. 2021 **[**16**]**		✓		✓	✓	
Cebotarenco & Bush 2008		✓		✓		
Chaintarli et al. 2016 **[**21**]**		✓		✓	✓	
Chan et al. 2021		✓			✓	
Farrell et al. 2011		✓			✓	
Fonseca et al. 2012	✓	✓	✓			✓
Formoso et al. 2013	✓	✓		✓	✓	
Francis et al. 2015		✓				
Gilham et al. 2023 **[**29**]**		✓		✓	✓	✓
Hale et al. 2017		✓	✓	✓	✓	
Hall et al. 2020 **[**30**]**		✓		✓	✓	
Hedin et al. 2006 [28]		✓	✓	✓		✓
Hogberg et al. 2004			✓			
Kesten et al. 2017		✓			✓	
Lecky et al. 2010		✓				
Lecky et al. 2017		✓			✓	
Madle et al. 2004		✓		✓		
Mazińska et al. 2017 [24]		✓	✓	✓	✓	
McNulty et al. 2001	✓	✓	✓		✓	✓
McNulty et al. 2010 [31]		✓			✓	
McNulty et al. 2020		✓			✓	
Munoz et al. 2014 **[**26**]**		✓	✓		✓	
Newitt et al. 2018 **[**23**]**		✓			✓	✓
Newitt et al. 2019		✓				
Parsons et al. 2004		✓	✓		✓	
Plachouras et al. 2014		✓		✓		
Pos-Doering et al. 2020	✓	✓			✓	
Rawson et al. 2018		✓		✓	✓	
Rönnerstrand et al. 2015 [25]		✓			✓	✓
Roope et al. 2020		✓		✓	✓	
Roque et al. 2016 **[**20**]**		✓			✓	
Sabuncu et al. 2009 **[**18**]**		✓			✓	
Scalas et al. 2017		✓	✓		✓	
van Hecke et al. 2019 [17]		✓			✓	
van Rijn et al. 2019		✓				
West & Cordina 2019		✓		✓		
Wilding et al. 2021		✓			✓	
Young et al. 2017		✓	✓	✓	✓	
Young et al. 2019		✓		✓	✓	
**Total no. of studies**	** 7**	**48**	**15**	**20**	**31**	**8**

a Reference numbers presented for only studies cited within the main paper. Full list of included study references is shown in Supplementary Table S2.

Individual-targeted interventions aimed to raise awareness, e.g. using leaflets sent from clinics or pharmacies to patients’ homes based on high-risk and vulnerability criteria [19, 20]. The individual-small group intervention approaches included training on hand hygiene, music and videos. School-based interventions include activities targeted at students and parents/guardians. At the population scale, the most common interventions were national campaigns to raise awareness. A particular example was the web-based Antibiotic Guardian (AG) campaign, which enabled people to register their pledges [21–23]. Interventions were delivered using face-to-face, online—including online games, such as e-Bug games—and hybrid formats.

### Effectiveness of AMR community interventions

The key findings related to intervention effectiveness and other related considerations are grouped under four overarching outcome categories as summarized below and presented in Supplementary Table S3.

#### Knowledge, awareness, and motivation

Generally, studies reported increased AMR knowledge and awareness after the interventions. A 21-year national media campaign showed an increase in awareness; however, although 93% reported knowing about antibiotics, 40% still believed they work against viruses [16]. Some interventions, such as the Antibiotic Guardian (AG) campaign, fostered social and personal responsibility, as reported with improved pledged commitment and action (30.7% to 63.4%), and increased a sense of personal responsibility towards tackling AMR from 58.3% to 70.5% [21]. Demographic discrepancies in beliefs about antibiotic effectiveness included perceived effectiveness of antibiotics against cold and flu being higher among males than females (OR = 2.16, 95%CI = 1.88–2.48) and (OR = 2.2, 95%CI = 1.91–2.54), respectively; higher in younger people aged 18–24 than older adults (e.g. flu ≥60 OR = 0.19, 95%CI = 0.13–0.28), and higher among those with lower education compared to those with higher level (e.g. flu OR = 3.53, 95%CI = 2.62–4.76) [24].

#### Changes in antibiotic use

Studies reported decreased antibiotic consumption rates in the intervention groups, especially among children and young adults, but the changes were not always statistically significant [18, 20]. A study from Poland showed reduced expectation to be prescribed antibiotics for cold [19% to 15%, (-4%, *P* = .019)] and flu [43% to 32%, (−11%, *P* < .001)] [24]. Another study reported increased willingness to postpone antibiotic treatment by up to 5 days (mean = 4.32 days; 95%CI = 4.05–4.58; *P* < .001) [25].

#### Changes in adherence

Most post-intervention evidence showed increased adherence and reduced non-compliance. Another controlled experiment in Spain reported a significant (18.8%) difference between the intervention vs control groups after the delivery of a patient antibiotic adherence education at a community pharmacy (67.2% vs 48.4%, 95%CI = 15.8–34.6, *P* = .033) and 43.1% reduced difference in non-compliance among those missing >1 dose (95%CI = 16.4–63.1%, *P* = .001) [26]. Another study in Malta showed that non-adherent patients with >2 tablets/capsules left were 9.2% lower among the intervention compared to the control group (70.0% vs 79.2%).

#### Changes in healthcare outcomes

Only a few studies reported on infection incidence. An educational and hand hygiene program in daycare centres and homes in Spain showed lower rates of respiratory infection episodes in the intervention compared to the control group (IRR = 0.77; 95%CI = 0.68–0.88) [27]. A study from Northern Ireland reported that although a revised antibiotic policy intervention reduced the MRSA incidence rate by 0.00561/100 bed-days per month, the change was not statistically significant in the hospital setting (*P* = .57), but showed a positive change in community MRSA incidence (*P* = .03) [19]. A daycare intervention reported a reduction in doctor consultations per child (RR = 0.81; 95%CI = 0.63–1.04) and a reduction in antibiotic prescriptions (RR = 0.70; 95%CI = 0.48–1.02) [28].

### Reach of interventions

Most studies which looked at interventions such as mass campaigns did not report intervention outcome data by specific sociodemographic (e.g. age, gender, ethnicity/race, and education). But a large number of interventions were aimed at school children or students at different levels of education. For example, the e-Bug online game-based intervention, implemented in several European countries, targeted primary and secondary school children aged 7–15 years.

Studies reporting data on web-based pledge campaigns found that reach extended to significant numbers, countries and population groups [22, 23, 29]. Reporting on the Keep Antibiotics Working campaign, Gilham et al. [21] stated that a UK campaign received over 10 million views on social media in its first year (2017/18) and 5.6 million views in its final year (2019/20). However, in another study, it was reported that 60.6% of registered AGs pledgers had not seen some of the promotional materials, especially members of the public (43.2% of the public vs 33.9% of healthcare professionals), possibly because the campaign was mainly promoted through healthcare settings and did not include reminders [21]. In another study, although many parents suggested TV adverts, websites, and social media may be good formats for campaigns, some parents felt these were impersonal without opportunity for dialogue, and instead they proposed face-to-face dissemination at playgroups (e.g. National Childbirth Trust groups) and other health contact opportunities (e.g. health visitors) [17].

Antibiotic consumption post-national campaign intervention in France was linked to those initially considered the biggest antibiotic consumers (adults 26–35 years old), and most parents of young children fall within this age category [18]. As a result, young adults were likely specifically affected by the campaign.

#### Long-term impact and sustainability

Only a few studies measured post-intervention changes longitudinally, with most studies reporting changes immediately after the intervention. Hall et al. [30] ran a second post-test questionnaire among students six months after the intervention and found knowledge gain to be sustained over this period. However, one study which looked at changes in people’s attitudes and use of antibiotics after one year of national campaigns in England and Scotland, found only a small increase in recollection of campaign posters (23.7% in 2009 vs 19.2% in 2008, *P* = .03), but no improvements in the understanding about lack of benefits of antibiotics use for coughs and colds [31]. The same study detected a significant increase in respondents retaining leftover antibiotics (2.2% in 2008—7.0% in 2009, *P* ≤ .001) [31]. The longest time gap between two evaluation points was reported in the 21-year period study from Belgium, and this showed less recall report (46% in 1997 vs 44.6% in 2018) [16].

## Discussions

We systematically evaluated community-based AMR interventions in Europe, and the included studies were from different community settings across 14 countries. The interventions were primarily educational and focused on raising awareness of antibiotic use and AMR. These were conducted at different population scales—individuals, small groups, or public—targeting specific groups or the general population. Individual and small group intervention approaches used the high-vulnerability (e.g. interventions for parents of young children) or opportunistic focus (e.g. school students as future citizens) and used approaches such as workshops, games, etc., while public campaigns were the most common intervention for the general population. Main outcomes of the community-based interventions included increased knowledge and awareness about antibiotics and AMR, willingness to postpone antibiotic treatment, adherence, lowering expectations of antibiotics, and reduction in consultations and prescriptions. A few interventions considered reported savings in cost and time. The evidence on the long-term effects of the interventions remains inconclusive, similar to other review findings [8].

In Europe, AMR in the community has increased during the last decades, and patients with AMR infections are more challenging to treat [32]. One of the major modifiable factors contributing to AMR is the inappropriate use and overuse of antimicrobials such as antibiotics [33]. So, from a public health perspective, identifying factors contributing to overuse and addressing these are urgently required [34]. Our review found that knowledge is the most commonly addressed factor by the interventions. Most interventions report immediate knowledge gain after an intervention, with only a few studies reporting long-term knowledge retention [30], and one reported loss of knowledge in the long run [31]. From a sustainability perspective, it is therefore essential for future interventions to consider, plan, and budget for retention strategies, such as curriculum integration, repeated messaging, and reminder systems, that extend beyond the immediate post-intervention period. Moreover, knowledge alone cannot lead to behavioural change, and interventions need to include elements that address behavioural components, such as motivation, and opportunities that are essential for a behaviour to take place. A recent scoping review on behaviour change interventions addressing antibiotic treatment-seeking behaviour among patients found that only a small portion of interventions targeted decision-making processes (4/38) or psychological drivers of antibiotic-seeking behaviour (3/38) [35].

A significant gap identified in this review is the lack of systematic sociodemographic reporting in most interventions. These details are important as previous research have shown demographic trends in antibiotic (mis)use. For example, older age, low level of education, ethnicity, migration status, etc., have been reported to be associated with inappropriate antibiotic use [35, 36]. Without disaggregated data, interventions risk reinforcing existing health inequalities and failing to address the disproportionate burden of AMR among certain demographic groups [37].

Use of community engagement to address this ‘super wicked problem’ is crucial in AMR interventions [38]. Previous research has suggested that community engagement in AMR management can facilitate AMR behaviour change because it is a contextualized approach which supports community needs [39]. Promoting community AMR interventions also enables individuals and populations to take ownership of their knowledge and actions, and co-develop meaningful solutions within their own settings [39]. Although there were positive changes in the identified interventions in this review, their effects were often small and not statistically significant. This may be because the specificity and context-dependency of community engagement can make it difficult to evaluate and scale the interventions or attribute changes to a single intervention.

Nevertheless, despite the low specificity potential of community interventions, they still have great scalability and potential for sustainable impact [39]. Hence, more evidence regarding the ability of community approaches to address AMR challenges is needed. Other considerations to be factored in include balancing the type of intervention with the location, target populations and preferred delivery medium [8, 17, 21]. Caution is also needed to avoid presumptions that an effective intervention in one context is likely to be effective in another because outcomes can be influenced by differences in healthcare organization, culture or country [39].

### Strengths and limitations

This review summarized interventions related to community-focused interventions aimed at reducing AMR. One strength was the description of various AMR interventions at different population scales across Europe. Limitations of the review include the search using only studies reported in the English language, which could have led to an overrepresentation of UK-based studies in the sample and excluded research studies in other languages and European nations. Setting the cut-off year at 2000 might have led to exclusion of interventions conducted pre-2000, and we only included peer-reviewed research which meant grey literature on the topic was missed. Also, we were unable to conduct in-depth statistical analysis due to the high heterogeneity between the outcomes reported across the studies. However, the inclusion of 14 countries demonstrates a broad representation from different countries in Europe.

### Implications for research and practice

AMR is a global problem that requires global action and nationally tailored, critical interventions for community and individual interventions. At the same time, our review highlights the difficulties of ascertaining the impact of these interventions (most of which are ad-hoc and measure only short-term outcomes) at population scale. A key recommendation is to use a combination of different intervention strategies based on the One Health approach [39] to identify and target the highest-priority pathogens in different locations [2]. For instance, tailoring interventions to address discrepancies and beliefs about antibiotics’ effectiveness against specific conditions (e.g. cold and flu) and considering different population groups (by gender and age) [39]. For this, systematic reporting of demographic differences in intervention evaluations, particularly mass campaigns, can help present a high-level picture of the impact of these interventions on different groups and identify those in need of greater support. Simultaneously, designing interventions that integrate One Health principles, like AMR education with food, pets, farming, and environmental hygiene are also needed [40].

AMR requires cross-sector stakeholder engagement in human, animal, and environmental health. The global nature of the problem warrants more surveillance, funding, capacity building, research and development, and pathogen-specific priority setting from the health community [2]. We, therefore, recommend the co-production of interventions with the community to encourage buy-in and ownership (acceptability) and community-generated solutions (feasibility) that facilitate two-way learning between community and intervention providers that improve scalability and sustainability [39]. To achieve this, further research is needed to understand the influence of culture and context on antibiotic use, as such factors may be a barrier to transferring effective interventions from one context/country to another.

## Conclusion

Our review presents a comprehensive synthesis of published evidence on the different types of community-based interventions initiated in Europe in the past 25 years with different population groups. We found that the majority of interventions were aimed at raising knowledge and awareness of appropriate antibiotic use and AMR, which were delivered through a range of strategies such as classroom teaching, theatre, pamphlets, web-based games, pledging, etc. While most interventions reported an increase in knowledge immediately after the intervention, evidence on the long-term sustainability of this knowledge is less clear, indicating that interventions might need to be consistently repeated for efficiency. Embedding antibiotic and AMR teaching in school curriculum or including programmes like eBug as part of regular classroom teaching can go a long way. Some interventions have measured changes in behaviour (adherence, expectation, prescribing), but these are often small-scale, localized studies that make generalization difficult. These behaviours are also closely linked with prescriber behaviour and hence joint interventions for patients and prescribers, that can build on cooperation, knowledge and trust among these two groups are needed. Further, large-scale and long-term interventions based on the One Health approach involving the community and assessment of behavioural outcomes would help understand what works and what does not, and in what contexts.

## Supplementary Material

ckaf257_Supplementary_Data

## Data Availability

The data underlying this article are available in the article and in its online supplementary material.
