# Supplementary material for: Community-based interventions for management of antimicrobial resistance in Europe: a systematic review
Source: Eur J Public Health. 2026 Jan 9;36(2):ckaf257. doi: 10.1093/eurpub/ckaf257 (PMC13064518; doi:10.1093/eurpub/ckaf257)
Supplement: ckaf257_Supplementary_Data [file ckaf257_supplementary_data.docx]

**Ekezie et al. Community-based interventions for management of antimicrobial resistance (AMR) in Europe: A systematic review**

# **Table S1 - AMR Community-based intervention SR Search (OVID Medline)**

1 Microbial Drug Resistance.mp. or exp Drug Resistance, Microbial/

2 Drug Resistance, Multiple/

3 exp Anti-Infective Agents/

4 (antimicrobial resist* OR anti microbial resist* or anti-microbial resist* or AMR).mp.

5 (multiresistant OR multiresistance OR "multi-resistant" OR "multi-resistance" OR MDR OR MDRO OR AMR).ti,ab

6 exp Community-Acquired Infections/

7 ((drug* OR multidrug OR “multi-drug” OR antimicrobial* OR “anti-microbial*” OR antiinfection* OR “anti-infection*” OR antibacterial* OR “anti-bacterial*” OR antibiotic* OR “anti-biotic*”) AND (resistant OR resistance OR tolerant OR tolerance)).ti,ab

8 (Methicillin-Resistant Staphylococcus aureus OR MRSA OR Gram-negative antibiotic resistance OR Escherichia coli OR Klebsiella pneumoniae OR Neisseria gonorrhoeae OR Mycobacterium tuberculosis OR drug-resistant HIV OR Plasmodium falciparum OR drug-resistant fungal infections OR Candida auris).mp.

9 Anti-Infective Agents

10 Anti-Bacterial Agents

11 1 or 2 or 3 or 4 or 5 or 6 or 7 or 8 or 9 or 10

12 exp Community Health Services/

13 exp Community Health Centers/

14 exp Community Medicine/

15 exp Community-Institutional Relations/

16 exp Hospitals, Community/

17 exp Health Education/

18 (Community Interven* OR Community service* OR Community care OR Community-based OR Community based OR Community level* OR Community acti* OR Community approach OR Community manag* OR community program* OR public health interven* OR public interven* OR public service* OR population interven*).mp

19 exp Persuasive Communication/

20 12 or 13 or 14 or 15 or 16 or 17 or 18 or 19

21 11 and 22

22 exp animals/ not humans.sh.

23 21 NOT 22

24 limit 23 to (english language and yr="2000 -Current")

# **Table S2 – References of the included studies (n=49)**

1. Ahmed R, Bashir A, Brown JEP, Cox JAG, Hilton AC, Jordan SL, et al. Aston University's Antimicrobial Resistance (AMR) Roadshow: raising awareness and embedding knowledge of AMR in key stage 4 learners. Infection Prevention in Practice. 2020; 2:100060.
2. Ahmed R, Bashir A, Brown JEP, Cox JAG, Hilton AC, Hilton CE, et al. The drugs don't work: evaluation of educational theatre to gauge and influence public opinion on antimicrobial resistance. Journal of Hospital Infection. 2020; 104:193-7.
3. Aldeyab MA, Scott MG, Kearney MP, Alahmadi YM, Magee FA, Conlon G, et al. Impact of an enhanced antibiotic stewardship on reducing methicillin-resistant Staphylococcus aureus in primary and secondary healthcare settings. Epidemiol Infect. 2014; 142:494-500.
4. Allison DG, Higginson P, Martin S. Antibiotic resistance awareness: a public engagement approach for all pharmacists. The International journal of pharmacy practice. 2017; 25:93-6.
5. Atkins L, Chadborn T, Bondaronek P, Ashiru-Oredope D, Beech E, Herd N, et al. Content and Mechanism of Action of National Antimicrobial Stewardship Interventions on Management of Respiratory Tract Infections in Primary and Community Care. Antibiotics (Basel, Switzerland). 2020; 9.
6. Azevedo M-M, Pinheiro C, Yaphe J, Baltazar F. Assessing the impact of a school intervention to promote students' knowledge and practices on correct antibiotic use. International journal of environmental research and public health. 2013; 10:2920-31.
7. Azor-Martinez E, Yui-Hifume R, Munoz-Vico FJ, Jimenez-Noguera E, Strizzi JM, Martinez-Martinez I, et al. Effectiveness of a Hand Hygiene Program at Child Care Centers: A Cluster Randomized Trial. Pediatrics. 2018; 142.
8. Barchitta M, Quattrocchi A, Maugeri A, Rosa MCL, Mastra CL, Basile G, et al. The "Obiettivo Antibiotico" Campaign on Prudent Use of Antibiotics in Sicily, Italy: The Pilot Phase. International journal of environmental research and public health. 2020; 17.
9. Bauraind I, Lopez-Lozano J-M, Beyaert A, Marchal J-L, Seys B, Yane F, et al. Association between antibiotic sales and public campaigns for their appropriate use. JAMA. 2004; 292:2468-70.
10. Bhattacharya A, Hopkins S, Sallis A, Budd EL, Ashiru-Oredope D. A process evaluation of the UK-wide Antibiotic Guardian campaign: developing engagement on antimicrobial resistance. Journal of public health (Oxford, England). 2017; 39:e40-e7.
11. Bruyndonckx R, Coenen S, Hens N, Vandael E, Catry B, Goossens H. Antibiotic use and resistance in Belgium: the impact of two decades of multi-faceted campaigning. Acta Clinica Belgica: International Journal of Clinical and Laboratory Medicine. 2021; 76:280-8.
12. Cebotarenco N, Bush PJ. Reducing antibiotics for colds and flu: a student-taught program. Health education research. 2008; 23:146-57.
13. Chaintarli K, Ingle SM, Bhattacharya A, Ashiru-Oredope D, Oliver I, Gobin M. Impact of a United Kingdom-wide campaign to tackle antimicrobial resistance on self-reported knowledge and behaviour change. BMC Public Health. 2016; 16:393.
14. Chan AHY, Horne R, Lycett H, Raebel E, Guitart J, Wildman E, et al. Changing patient and public beliefs about antimicrobials and antimicrobial resistance (AMR) using a brief digital intervention. Frontiers in Pharmacology. 2021; 12:608971.
15. Farrell D, Kostkova P, Weinberg J, Lazareck L, Weerasinghe D, Lecky DM, et al. Computer games to teach hygiene: an evaluation of the e-Bug junior game. J Antimicrob Chemother. 2011; 66 Suppl 5:v39-44.
16. Fonseca MJ, Santos CL, Costa P, Lencastre L, Tavares F. Increasing awareness about antibiotic use and resistance: a hands-on project for high school students. PloS one. 2012; 7:e44699.
17. Formoso G, Paltrinieri B, Marata AM, Gagliotti C, Pan A, Moro ML, et al. Feasibility and effectiveness of a low cost campaign on antibiotic prescribing in Italy: community level, controlled, non-randomised trial. BMJ (Clinical research ed). 2013; 347:f5391.
18. Francis N. A local public campaign reduces outpatient antibiotic prescribing in Italy. Evidence-based nursing. 2015; 18:27.
19. Gilham EL, Casale E, Hardy A, Ayeni AH, Sunyer E, Harris T, et al. Assessing the impact of a national social marketing campaign for antimicrobial resistance on public awareness, attitudes, and behaviour, and as a supportive tool for healthcare professionals, England, 2017 to 2019. Euro surveillance : bulletin Europeen sur les maladies transmissibles = European communicable disease bulletin. 2023; 28.
20. Hale AR, Young VL, Grand A, McNulty CAM. Can gaming increase antibiotic awareness in children? A mixed-methods approach. JMIR Serious Games. 2017; 5:e6420.
21. Hall J, Jones L, Robertson G, Hiley R, Nathwani D, Perry MR. 'The Mould that Changed the World': Quantitative and qualitative evaluation of children's knowledge and motivation for behavioural change following participation in an antimicrobial resistance musical. PloS one. 2020; 15:e0240471.
22. Hedin K, Petersson C, Cars H, Beckman A, Hakansson A. Infection prevention at day-care centres: feasibility and possible effects of intervention. Scandinavian journal of primary health care. 2006; 24:44-9.
23. Hogberg L, Henriques Normark B, Ringberg H, Stenqvist K, Fredlund H, Geli P, et al. The impact of active intervention on the spread of penicillin-resistant Streptococcus pneumoniae in Swedish day-care centres. Scandinavian journal of infectious diseases. 2004; 36:629-35.
24. Kesten JM, Bhattacharya A, Ashiru-Oredope D, Gobin M, Audrey S. The Antibiotic Guardian campaign: a qualitative evaluation of an online pledge-based system focused on making better use of antibiotics. BMC public health. 2017; 18:5.
25. Lecky DM, McNulty CAM, Touboul P, Herotova TK, Benes J, Dellamonica P, et al. Evaluation of e-Bug, an educational pack, teaching about prudent antibiotic use and hygiene, in the Czech Republic, France and England. The Journal of antimicrobial chemotherapy. 2010; 65:2674-84.
26. Lecky DM, Dhillon H, Verlander NQ, McNulty CAM. Animations designed to raise patient awareness of prudent antibiotic use: patient recall of key messages and their immediate effect on patient attitude. BMC research notes. 2017; 10:701.
27. Madle G, Kostkova P, Mani-Saada J, Weinberg J, Williams P. Changing public attitudes to antibiotic prescribing: can the internet help? Journal of Innovation in Health Informatics. 2004; 12:19-26.
28. Mazińska B, Strużycka I, Hryniewicz W. Surveys of public knowledge and attitudes with regard to antibiotics in Poland: Did the European Antibiotic Awareness Day campaigns change attitudes? PLoS One. 2017; 12:e0172146.
29. McNulty CAM, Swan AV, Boland D. Schools’ antimicrobial resistance: National Advice to the Public campaign–a pilot study. Health Education. 2001; 101:235-42.
30. McNulty CAM, Nichols T, Boyle PJ, Woodhead M, Davey P. The English antibiotic awareness campaigns: did they change the public's knowledge of and attitudes to antibiotic use? The Journal of antimicrobial chemotherapy. 2010; 65:1526-33.
31. McNulty CAM, Syeda RB, Brown CL, Bennett CV, Schofield B, Allison DG, et al. Peer-education as a tool to educate on antibiotics, resistance and use in 16–18-year-olds: A feasibility study. Antibiotics. 2020; 9:146.
32. Munoz EB, Dorado MF, Guerrero JE, Martinez FM. The effect of an educational intervention to improve patient antibiotic adherence during dispensing in a community pharmacy. Atencion primaria. 2014; 46:367-75.
33. Newitt S, Anthierens S, Coenen S, Lo Fo Wong D, Salvi C, Puleston R, et al. Expansion of the 'Antibiotic Guardian' one health behavioural campaign across Europe to tackle antibiotic resistance: pilot phase and analysis of AMR knowledge. European journal of public health. 2018; 28:437-9.
34. Newitt S, Oloyede O, Puleston R, Hopkins S, Ashiru-Oredope D. Demographic, Knowledge and Impact Analysis of 57,627 Antibiotic Guardians Who Have Pledged to Contribute to Tackling Antimicrobial Resistance. Antibiotics (Basel, Switzerland). 2019; 8.
35. Parsons S, Morrow S, Underwood M. Did local enhancement of a national campaign to reduce high antibiotic prescribing affect public attitudes and prescribing rates? The European journal of general practice. 2004; 10:18-23.
36. Plachouras D, Antoniadou A, Giannitsioti E, Galani L, Katsarolis I, Kavatha D, et al. Promoting prudent use of antibiotics: the experience from a multifaceted regional campaign in Greece. BMC public health. 2014; 14:866.
37. Pos-Doering R, Kuehn L, Kamradt M, Glassen K, Fleischhauer T, Kaufmann-Kolle P, et al. Converting habits of antibiotic use for respiratory tract infections in German primary care (CHANGE-3) - process evaluation of a complex intervention. BMC family practice. 2020; 21:274.
38. Rawson TM, Moore LSP, Castro-Sanchez E, Charani E, Hernandez B, Alividza V, et al. Development of a patient-centred intervention to improve knowledge and understanding of antibiotic therapy in secondary care. Antimicrobial resistance and infection control. 2018; 7:43.
39. Rönnerstrand B, Andersson Sundell K. Trust, reciprocity and collective action to fight antibiotic resistance. An experimental approach. Soc Sci Med. 2015; 142:249-55.
40. Roope LSJ, Tonkin-Crine S, Herd N, Michie S, Pouwels KB, Castro-Sanchez E, et al. Reducing expectations for antibiotics in primary care: a randomised experiment to test the response to fear-based messages about antimicrobial resistance. BMC medicine. 2020; 18:110.
41. Roque F, Teixeira-Rodrigues A, Breitenfeld L, Piñeiro-Lamas M, Figueiras A, Herdeiro MT. Decreasing antibiotic use through a joint intervention targeting physicians and pharmacists. Future Microbiol. 2016; 11:877-86.
42. Sabuncu E, David J, Bernède-Bauduin C, Pépin S, Leroy M, Boëlle PY, et al. Significant reduction of antibiotic use in the community after a nationwide campaign in France, 2002-2007. PLoS Med. 2009; 6:e1000084.
43. Scalas D, Roana J, Mandras N, Cuccu S, Banche G, Marra ES, et al. The Microbiological@mind project: a public engagement initiative of Turin University bringing microbiology and health education into primary schools. International journal of antimicrobial agents. 2017; 50:588-92.
44. Van Hecke O, Butler CC, Wang K, Tonkin-Crine S. Parents' perceptions of antibiotic use and antibiotic resistance (PAUSE): a qualitative interview study. J Antimicrob Chemother. 2019; 74:1741-7.
45. van Rijn M, Haverkate M, Achterberg P, Timen A. The public uptake of information about antibiotic resistance in the Netherlands. Public understanding of science (Bristol, England). 2019; 28:486-503.
46. West LM, Cordina M. Educational intervention to enhance adherence to short-term use of antibiotics. Research in social & administrative pharmacy : RSAP. 2019; 15:193-201.
47. Wilding S, Kettu V, Thompson W, Howard P, Jeuken LJC, Pownall M, et al. Development and randomized controlled trial of an animated film aimed at reducing behaviours for acquiring antibiotics. JAC-Antimicrobial Resistance. 2021; 3:dlab083.
48. Young VL, Cole A, Lecky DM, Fettis D, Pritchard B, Verlander NQ, et al. A mixed-method evaluation of peer-education workshops for school-aged children to teach about antibiotics, microbes and hygiene. The Journal of antimicrobial chemotherapy. 2017; 72:2119-26.
49. Young VL, Berry M, Verlander NQ, Ridgway A, McNulty CAM. Using debate to educate young people in schools about antibiotic use and resistance: A before and after evaluation using a questionnaire survey. Journal of Infection Prevention. 2019; 20:281-8.

# **Table S3 - Summary of AMR community interventions outcome effectiveness**

| **Outcome Categories** | **Effectiveness** | **Other considerations** |
| --- | --- | --- |
| Knowledge, awareness and motivation | - Increased social responsibility and acceptance of personal responsibility [1, 2] - Increased clarification of confusion that improved commitment and action [2] - Knowledge level increase after watching an educational video significant for the least knowledgeable group [3] | - Continued believe antibiotic work against viruses [4]. - Demographic discrepancies: Perceived antibiotics effectiveness against cold and flu higher among males, younger people, and those with lower education) [5] - Post-intervention knowledge decrease in intervention group, although prescribing rates decreased, indicating changes in practitioner behaviour [6] - Observed improvements in knowledge and understanding were considered short-term [7]. |
| Changes in antibiotic use | - Substantial reduction of antibiotic use in children 6–15 years old and young adults 26–35 years old [8]. - Decrease in outpatient antibiotic use [4]. - Antibiotic costs fluctuation but eventual decreased, resulting in cost savings [4]. - Reduced expectancy for antibiotics for bronchitis, flu, sore throat, common cold and diarrhoea [4, 5]. - Increased willingness to postpone antibiotic treatment [9]. | - Antibiotic consumption changes in the intervention groups not always statistically significant [8, 10]. |
| Changes in adherence | - Reduced difference in non-compliance among those missing recommended doses [11, 12]. - Reduced cost of wasted antibiotics from use of educational leaflets [12]. | - No significant differences were found in patient-perceived health [11]. - General overuse beliefs associated with non-adherence [12]. - Generalised trust associated with a willingness to postpone antibiotic treatment [9]. - After adherence education at a community pharmacy no significant differences found in patient-perceived health [11] - Predictors of adherence: adequate medication knowledge, co-incidence between the duration of treatment indicated by the physician and duration of treatment written in the prescription [11]. |
| Changes in healthcare outcomes | - Lower rates of respiratory infection episodes in the intervention [13]. - Reduced MRSA incidence in community [14]. - Reduction in doctor consultations and antibiotic prescriptions [15]. - Immediate antibiotic prescription possibly reduced the duration of moderately bad symptoms, but not offering antibiotics or using delayed prescribing were also acceptable to most patients [16]. | - Reduced MRSA incidence not statistically significant in the hospital setting compared to community [14]. - . |

**Included references mention in Table S3**

[1] O. Van Hecke, C. C. Butler, K. Wang, and S. Tonkin-Crine, "Parents' perceptions of antibiotic use and antibiotic resistance (PAUSE): a qualitative interview study," *J Antimicrob Chemother,* vol. 74, no. 6, pp. 1741-1747, 2019, doi: 10.1093/jac/dkz091.

[2] K. Chaintarli, S. M. Ingle, A. Bhattacharya, D. Ashiru-Oredope, I. Oliver, and M. Gobin, "Impact of a United Kingdom-wide campaign to tackle antimicrobial resistance on self-reported knowledge and behaviour change," *BMC Public Health,* vol. 16, p. 393, 2016, doi: 10.1186/s12889-016-3057-2.

[3] M. van Rijn, M. Haverkate, P. Achterberg, and A. Timen, "The public uptake of information about antibiotic resistance in the Netherlands," *Public understanding of science (Bristol, England),* vol. 28, no. 4, pp. 486-503, 2019, doi: <https://dx.doi.org/10.1177/0963662518823701>.

[4] R. Bruyndonckx, S. Coenen, N. Hens, E. Vandael, B. Catry, and H. Goossens, "Antibiotic use and resistance in Belgium: the impact of two decades of multi-faceted campaigning," *Acta Clinica Belgica: International Journal of Clinical and Laboratory Medicine,* vol. 76, no. 4, pp. 280-288, 2021, doi: 10.1080/17843286.2020.1721135.

[5] B. Mazińska, I. Strużycka, and W. Hryniewicz, "Surveys of public knowledge and attitudes with regard to antibiotics in Poland: Did the European Antibiotic Awareness Day campaigns change attitudes?," *PLoS One,* vol. 12, no. 2, p. e0172146, 2017, doi: 10.1371/journal.pone.0172146.

[6] G. Formoso *et al.*, "Feasibility and effectiveness of a low cost campaign on antibiotic prescribing in Italy: community level, controlled, non-randomised trial," *BMJ (Clinical research ed.),* vol. 347, no. 8900488, bmj, 101090866, p. f5391, 2013, doi: <https://dx.doi.org/10.1136/bmj.f5391>.

[7] T. M. Rawson *et al.*, "Development of a patient-centred intervention to improve knowledge and understanding of antibiotic therapy in secondary care," *Antimicrobial resistance and infection control,* vol. 7, no. 101585411, p. 43, 2018, doi: <https://dx.doi.org/10.1186/s13756-018-0333-1>.

[8] E. Sabuncu *et al.*, "Significant reduction of antibiotic use in the community after a nationwide campaign in France, 2002-2007," *PLoS Med,* vol. 6, no. 6, p. e1000084, 2009, doi: 10.1371/journal.pmed.1000084.

[9] B. Rönnerstrand and K. Andersson Sundell, "Trust, reciprocity and collective action to fight antibiotic resistance. An experimental approach," *Soc Sci Med,* vol. 142, pp. 249-55, 2015, doi: 10.1016/j.socscimed.2015.08.032.

[10] F. Roque, A. Teixeira-Rodrigues, L. Breitenfeld, M. Piñeiro-Lamas, A. Figueiras, and M. T. Herdeiro, "Decreasing antibiotic use through a joint intervention targeting physicians and pharmacists," *Future Microbiol,* vol. 11, pp. 877-86, 2016, doi: 10.2217/fmb-2016-0010.

[11] E. B. Munoz, M. F. Dorado, J. E. Guerrero, and F. M. Martinez, "The effect of an educational intervention to improve patient antibiotic adherence during dispensing in a community pharmacy," *Atencion primaria,* vol. 46, no. 7, pp. 367-75, 2014, doi: <https://dx.doi.org/10.1016/j.aprim.2013.12.003>.

[12] L. M. West and M. Cordina, "Educational intervention to enhance adherence to short-term use of antibiotics," *Research in social & administrative pharmacy : RSAP,* vol. 15, no. 2, pp. 193-201, 2019, doi: <https://dx.doi.org/10.1016/j.sapharm.2018.04.011>.

[13] E. Azor-Martinez *et al.*, "Effectiveness of a Hand Hygiene Program at Child Care Centers: A Cluster Randomized Trial," *Pediatrics,* vol. 142, no. 5, 2018, doi: <https://dx.doi.org/10.1542/peds.2018-1245>.

[14] M. A. Aldeyab *et al.*, "Impact of an enhanced antibiotic stewardship on reducing methicillin-resistant Staphylococcus aureus in primary and secondary healthcare settings," *Epidemiol Infect,* vol. 142, no. 3, pp. 494-500, 2014, doi: 10.1017/S0950268813001374.

[15] K. Hedin, C. Petersson, H. Cars, A. Beckman, and A. Hakansson, "Infection prevention at day-care centres: feasibility and possible effects of intervention," *Scandinavian journal of primary health care,* vol. 24, no. 1, pp. 44-9, 2006.

[16] P. Little *et al.*, "Information leaflet and antibiotic prescribing strategies for acute lower respiratory tract infection: a randomized controlled trial," *JAMA,* vol. 293, no. 24, pp. 3029-35, 2005, doi: <https://dx.doi.org/10.1001/jama.293.24.3029>.
